# The Degree of Menstrual Disturbance Is Associated With the Severity of Insulin Resistance in PCOS

**DOI:** 10.3389/fendo.2022.873726

**Published:** 2022-06-13

**Authors:** Xiaojia Li, Dongyong Yang, Ping Pan, Ricardo Azziz, Dongzi Yang, Yanxiang Cheng, Xiaomiao Zhao

**Affiliations:** ^1^ Department of Obstetrics and Gynecology, Sun Yat-sen Memorial Hospital, Sun Yat-sen University, Guangzhou, China; ^2^ Department of Obstetrics and Gynecology, Renmin Hospital of Wuhan University, Wuhan, China; ^3^ Department of Obstetrics & Gynecology, School of Medicine, University of Alabama at Birmingham, Birmingham, AL, United States; ^4^ Department of Obstetrics & Gynecology, David Geffen School of Medicine, University of California Los Angeles, Los Angeles, CA, United States; ^5^ Department of Health Policy, Management and Behavior, School of Public Health, University at Albany, State University of New York (SUNY), Albany, NY, United States; ^6^ Center for Reproductive Medicine, Department of Obstetrics and Gynecology, Guangdong Provincial People’s Hospital, Guangdong Academy of Medical Sciences, Guangzhou, China

**Keywords:** polycystic ovary syndrome, glucose metabolism, insulin resistance, vaginal bleeding intervals, hyperandrogenism

## Abstract

**Objective:**

Insulin resistance (IR) is an important determinant of the phenotype and morbidity of the polycystic ovary syndrome (PCOS). In this study, we aimed to figure out the association between the degree of menstrual disturbance and the severity of IR in women with PCOS.

**Design:**

It is a cross-sectional study conducted in an academic tertiary setting.

**Patients:**

The patients comprised five hundred twenty-seven women diagnosed with PCOS by the 2003 Rotterdam criteria and 565 controls with regular vaginal bleeding.

**Interventions:**

The interventions done for this study are medical history collection, physical examination, and blood sampling.

**Main outcome measures:**

The main outcome measures are body mass index (BMI), fasting glucose, fasting insulin, homeostatic model assessment for IR (HOMA-IR), and hormonal parameters.

**Results:**

Women with PCOS had a higher level of BMI, HOMA-IR, and HOMA-β than controls, with a decreased level of sex hormone-binding globulin and QUICK I index. The luteinizing hormone (LH)/follicle-stimulating hormone (FSH), testosterone (T), antral follicle count (AFC), dehydroepiandrosterone sulfate, free androgen index, modified Ferriman–Gallwey score, and the incidence of delayed insulin peak increased with the degree of menstrual disturbance, although there was no significance for the latter four parameters. Women with vaginal bleeding intervals of 45–90 days had a relatively higher level of HOMA-IR and HOMA-β, although it was adjusted with age and BMI than the other two groups. Similar results were observed in AUCI (area under the curve of insulin) and I/G [the ratio of AUCI and AUCG (area under the curve of glucose)]. Anovulatory women with vaginal bleeding episodes of less than 45 days tended to have higher glucose and insulin levels, area under the curve of glucose (AUCG), area under the curve of insulin (AUCI), HOMA-IR, and HOMA-β but decreased QUICK I and Matsuda index than those who were ovulatory. Women with vaginal bleeding intervals of longer than 45 days who had hyperandrogenism (HA) showed a higher level of glucose, insulin, HOMA-IR, and HOMA-β but lower QUICK I and Matsuda Index.

**Conclusions:**

In women with PCOS, the severity of IR, the LH/FSH ratio, and androgen level increased with a higher degree of disturbance in menstrual cyclicity (i.e., the vaginal bleeding intervals). Subgroup analysis indicated that the situation of HA may aggravate the disorder of glucose metabolism in women with PCOS. Overall, the interval between episodes of vaginal bleeding may be useful as a ready measure for predicting the severity of IR in PCOS.

## Introduction

Polycystic ovary syndrome (PCOS), characterized by hyperandrogenism (HA), ovulation dysfunction, and polycystic ovarian changes (1), is one of the most common endocrine and metabolic diseases of reproductive-aged women (2). Approximately 70% of women with PCOS are reported to be accompanied with insulin resistance (IR), which will further result in reproductive and metabolic complications in the long term (3–8). Therefore, identifying clinical and/or biological indicators to detect the early IR in women with PCOS will, to some extent, reduce the incidence of diabetes and metabolic syndrome and improve the life quality and long-term prognosis.

Oligomenorrhea and irregular menstrual cycle are important characteristics and criteria of PCOS, regardless of how PCOS is defined (9). Approximately 85%–90% of women with PCOS demonstrated oligoovulation and a prolonged interval between episodes of vaginal bleeding (9, 10). A cohort study indicated that irregular and long menstrual cycles have a strong correlation with hyperinsulinemia (8); these observations are in accord with the results from a cross-sectional study, in which the authors found that women with 35-day-longer bleeding intervals had a higher level of homeostatic model assessment for IR (HOMA-IR) than healthy controls, suggesting that the severity of oligomenorrhea is likely to be positively correlated with IR in PCOS (11). These studies revealed that menstrual dysfunction could be applied as an effective clinical marker to evaluate the potential metabolic disorders in women with PCOS. However, these studies are focused on the association between irregular menstrual cycles and the risk of type 2 diabetes mellitus in women from Spain and the United States. There are limited studies exploring the correlation between menstrual disturbance and the severity of IR in PCOS subjects who are anovulatory and with hyperandrogenism (HA). Given the heterogeneity of PCOS and racial difference, a cross-sectional study was conducted to evaluate the relationship between the degree of menstrual irregularity and glucose metabolic dysfunction in women with PCOS of Asian populations. The influence of the ovulatory status and serum androgen level on glucose metabolism was also considered.

In this study, a total of 527 women with PCOS and 565 controls were recruited. We evaluated the correlation between the menstrual cycle and body mass index (BMI), fasting glucose, fasting insulin, HOMA-IR, triglycerides, and other indicators. Our results revealed that the degree of menstrual dysfunction could act as a robust clinical marker to predict the severity of IR in women with PCOS.

## Materials and Methods

### Subjects

Five hundred twenty-seven women with PCOS and 565 controls were recruited from patients presented to the Reproductive Medicine Center of Sun Yat-sen Memorial Hospital, Sun Yat-sen University between 2009 and 2015. PCOS was diagnosed by the Rotterdam 2003 criteria and was defined by the presence of either two of the following three features: 1) oligo- or anovulation, 2) clinical and/or biochemical signs of hyperandrogenism, and 3) the presence of polycystic ovarian morphology under ultrasound (12). PCOS was diagnosed only after other related disorders had been excluded.

Women with infertility caused by fallopian tube obstruction or male factors (asthenozoospermia or azoospermia) were recruited as control. All control subjects had a long-term history of regular vaginal bleeding (26–35 days) consistent with ovulatory cycles, did not have polycystic ovarian morphology on ultrasonography, and were non-hirsute [modified Ferriman–Gallwey (mFG) score ≤ 3] (13). Controls were excluded if the detailed information of the vaginal bleeding interval was unavailable or if they received a hormonal medication within 3 months of evaluation.

Related disorders were excluded by assessing thyroid stimulating hormone (TSH), prolactin, 17-hydroxyprogesterone, and so on. Screening for Cushing’s syndrome and androgen-secreting neoplasms was performed if clinically indicated.

Subjects were eligible for inclusion with data available for the main outcome measurement if they could be categorized as either PCOS or controls. This study was approved by the Ethics Committee of Sun-Yat Sun Memorial Hospital of Sun Yat-Sen University, under the Chinese Clinical Trial Registry (https://www.chictr.org.cn/enIndex.aspx) number ChiCTR‐DDT‐14005186. All subjects signed written informed consent.

### Protocol

All subjects completed a questionnaire for personal information, menstrual history, relative family history, skin problems (hirsutism, acne, and premature alopecia) associated with hyperandrogenism, and metabolic diseases. Then, participants underwent a thorough medical evaluation including a physical exam for height, weight, waist and hip measurements, and transvaginal pelvic ultrasound (Philips EnVisor C HD Ultrasound) for antral follicle counting. Polycystic ovarian morphology (PCOM) was defined as 12 or more cysts measuring 2–9 mm in one side of the ovary and/or over 10 ml of either ovary volume (14). The mFG score was used to assess hair growth, and hirsutism was defined as an mFG score of ≥6 (15). All patients were examined and assigned an mFG score by XZ.

Women with PCOS were divided into three groups according to the vaginal bleeding intervals and classified as less than 45 days, 45–90 days, and longer than 90 days. Part of women with less than 45 days of bleeding intervals were eumenorrheic, and the ovulatory function was evaluated by the progesterone level in days 22–24 of menstrual cycle. Less than 4 ng/ml was considered as anovulatory, and the remainder were considered ovulatory (11). To study the correlation between ovulatory function and IR, women with vaginal bleeding intervals of less than 45 days were further divided as ovulatory and anovulatory groups. Fasting baseline blood samples for biochemical and hormone testing were obtained at the follicular or preovulatory phase of the cycle (days 2–4 of menstrual cycle) or at random for those who have amenorrhea. Participants were asked not to take any food except water for at least 8 h before testing. Glucose and total cholesterol (CHOL), triglyceride (TG), low-density lipoprotein (LDL), and high-density lipoprotein (HDL) were measured by a Beckman AU5800 automatic biochemical analyzer (Beckman Coulter, California, United States). The insulin level, dehydroepiandrosterone sulfate (DHEAS), and sex hormone-binding globulin (SHBG) were assessed by the automatic chemical luminescence immunoassay (Immulite 1000; Siemens, China Medical Solution Group). Serum hormones including the follicle-stimulating hormone (FSH), luteinizing hormone (LH), estradiol (E2), total testosterone (TT), and free testosterone (FT) were measured using Access 2 chemiluminescence immunoassays (Beckman, Chaska, MN, USA) according to the manufacturer’s protocols. The free androgen index (FAI) was calculated using the following formula: [TT (nmol/L) × 100/SHBG (nmol/L)].

### Evaluation of Insulin Resistance

The oral glucose tolerance test (OGTT) and insulin tolerance test (ITT) are currently the gold standard to evaluate glucose metabolism by assessing venous plasma glucose and the insulin level in a fasting state and 1 and 2 h after administering a glucose solution. The following mathematical models were used to evaluate IR. The homeostatic model for the assessment of insulin resistance (HOMA-IR) was calculated as follows (16): fasting glucose (mmol/L) × fasting insulin (μU/ml)/22.5. The homeostatic model for the assessment of β-cell function (HOMA-β) was calculated using the following formula (16): 20 × fasting insulin (μU/ml)/[fasting glucose (mmol/L) − 3.5]. The quantitative insulin sensitivity index (QUICK I) was calculated as follows (17): 1/[log(fasting insulin)(μU/ml) + log(fasting glucose)(mg/dl)]. The Matsuda insulin sensitivity index (Matsuda index) was calculated using the following formula (18): 10,000/√(fasting glucose (mg/dl) × fasting insulin (μU/ml) × mean glucose concentration (mg/dl) × mean insulin concentration (μU/ml).

### Statistical Analysis

Descriptive statistics were expressed as mean ± standard error. The ANOVA test was used to compare mean values among different groups. The χ2 test and Fisher’s exact test were used to compare categorical variables. A stepwise multiple regression analysis was used to compare the mean HOMA-IR, HOMA-β, QUICK I, and Matsuda index of controls with each cycle length group while controlling for BMI and age. Statistical analysis was performed using SPSS ver. 25.0 (SPSS, Inc., Chicago, IL, USA). *P <*0.05 was considered statistically significant.

## Results

### Basic Characteristics of Controls and Polycystic Ovary Syndrome Subjects With Different Vaginal Bleeding Intervals

A total of 527 PCOS patients and 565 controls were included in our analysis. The basic characteristics of the two groups are shown in **Table 1**. As a whole, women with PCOS had significantly higher BMI, LH/FSH, T, AFC, mFG, and FAI than those of controls, while the level of SHBG was significantly decreased in women with PCOS. Compared with the control group, PCOS subjects also had higher levels of fasting insulin, HOMA-IR, HOMA-β, CHOL, TGs, HDL, and LDL but lower levels of fasting glucose and QUICK I. These results suggested that PCOS is a heterogeneous disorder with multiple phenotypes, but the core pathogenesis of PCOS was IR and hyperandrogenism (HA).

**Table 1:** Basic characteristic of patients, glucose and lipid metabolism parameters between control and PCOS group.

	Control (N = 565)	All PCOS subjects (N = 527)	*P* value
Age (y)	26.00±3.59	25.93±5.05	0.809
BMI (kg/*m* ^2^)	20.35±2.58	22.69±4.17	<0.05
WHR	0.80±0.06	0.82±0.07	0.143
LH/FSH	0.56±0.41	1.71±1.22	<0.05
T (mmol/L)	1.34±0.62	2.24±1.02	<0.05
AFC	12.05±4.41	26.14±8.29	<0.05
FAI	2.39 (1.64-3.71)	4.28 (2.48-7.29)	<0.05
mFG	1.00 (0.00-2.00)	4.00 (2.00-8.00)	<0.05
SHBG (nmol/L)	62.60 (46.10-83.10)	50.91 (29.86-76.60)	<0.05
Fasting glucose (mmol/L)	5.17±0.56	5.11±0.53	<0.05
Fasting insulin (mU/ml)	3.12 (2.00-5.14)	8.02 (4.50-13.10)	<0.05
HOMA-IR	0.70 (0.47-1.19)	1.79 (0.99-2.89)	<0.05
HOMA-β (%)	40.00 (25.85-68.73)	103.64 (58.61-164.29)	<0.05
QUICK I	0.83 (0.69-0.98)	0.62 (0.55-0.74)	<0.05
CHOL (mmol/L)	4.57±1.09	4.99±0.92	<0.05
TG (mmol/L)	0.98±0.56	1.34±0.85	<0.05
HDL (mmol/L)	1.49±0.40	1.55±0.38	<0.05
LDL (mmol/L)	2.38±0.74	2.93±0.82	<0.05

BMI, body mass index; WHR, waist and hip ratio; FSH, follicle stimulating hormone; LH, luteinizing hormone; T, testosterone; AFC, antral follicle count; FAI, Free androgen index; mFG, Modified Ferriman-Gallwey score; SHBG, sex hormone binding globulin; HOMA-IR, homeostatic model for assessment of insulin resistance; HOMA-β, homeostatic model for assessment of β-cell function; QUICK I, quantitative insulin sensitivity index; CHOL, Cholesterol; TG, Triglyceride; HDL, High density lipoprotein; LDL, Low density lipoprotein.

Women with PCOS were then grouped based on the vaginal bleeding interval (**Table 2**). Seventy-three percent of PCOS subjects had apparent oligomenorrhea with the interval between episodes of vaginal bleeding of at least 45 days. Women with 45–90-day bleeding intervals comprised 50% of the total, 27% had bleeding intervals of less than 45 days, and the remaining 23% had longer than 90 days. As the intervals of vaginal bleeding were prolonged, the values of LH/FSH, T, DHEAs, AFC, FAI, and mFG tended to increase, although no statistically significant differences were found among groups for DHEAs, FAI, and mFG. There were no differences in age, BMI, waist-to-hip ratio, fasting glucose, and 1 and 2 h blood glucose values among groups. In contrast, both the fasting insulin level and 1 h blood insulin value increased in women with 45–90-day bleeding intervals but insulin value went down to a similar level to the other groups after 2 h of glucose load. Moreover, we noticed a phenomenon that the incidence of insulin peak delay tended to increase as vaginal bleeding intervals were prolonged. After adjusting for age and the BMI, PCOS subjects with a menstrual cycle of 45–90 days showed the highest HOMA-IR and HOMA-β but lowest Matsuda index; meanwhile, these women showed the highest levels of CHOL and LDL and the lowest levels of TG and HDL, although there was no statistical significance among groups (**Table 2**).

**Table 2:** Basic characteristic of patients, glucose and lipid metabolism parameters among groups of different menstrual cycle length.

	<45 days (N = 141)	45-90 days (N = 264)	>90 days (N = 122)	*P* value
Age (y)	26.33±5.50	25.91±5.06	25.53±4.47	0.437
BMI (kg/m^2^)	22.76±4.32	22.71±3.97	22.58±4.38	0.946
WHR	0.83±0.07	0.82±0.07	0.81±0.07	0.304
LH/FSH	1.50 ±1.46^c^	1.69±1.00	2.01±1.31^a^	<0.05
T (mmol/L)	2.05±0.99	2.20±1.02	2.55±0.98	<0.05
DHEAS (μg/dl)	2066.37±901.48	2112.97±1027.29	2263.85±1018.03	0.260
AFC	24.90±6.98^c^	25.91±7.93	28.03±9.98^a^	<0.05
FAI	3.76 (1.98-6.09)	4.50 (2.68-7.61)	4.82 (2.84-7.96)	0.109
mFG	3.00 (2.00-8.00)	4.00 (2.00-7.00)	5.00 (2.00-8.00)	0.198
Fasting glucose (mmol/L)	5.17±0.57	5.11±0.52	5.04±0.49	0.155
1h glucose (mmol/L)	8.17±2.57	8.34±2.18	8.20±2.32	0.727
2h glucose (mmol/L)	6.70±1.91	6.80±1.78	6.59±1.60	0.567
Fasting insulin (mU/ml)	8.06 (4.71-13.11)	8.06 (5.20-13.90)^c^	7.14 (3.81-11.60)^b^	<0.05
1h insulin (mU/ml)	77.60 (51.50-123.00)^b^	82.80 (54.95-136.25)^ac^	77.00 (50.40-125.00)^b^	<0.05
2h insulin (mU/ml)	68.60 (41.70-103.00)	65.20 (40.75-132.75)	67.20 (42.20-120.00)	0.086
Delayed insulin peak (%)	34.75 (49/141)	35.61 (94/264)	40.16 (49/122)	0.601
AUCG	13.65 (11.65-15.85)	13.48 (11.81-15.79)	14.30 (12.15-16.25)	0.744
AUGI	124.35 (78.95-177.74)^b^	125.95 (83.83-207.14)^ac^	119.29 (76.50-192.57)^b^	<0.05
I/G	8.63 (6.25-12.62)^b^	8.70 (6.21-13.91)^a^	8.29 (6.51-13.21)	<0.05
HOMA-IR	1.88 (1.10-3.02)	1.80 (1.12-2.96)^c^	1.59 (0.83-2.66)^b^	<0.05
Adjusted HOMA-IR	1.83 (1.02-2.97)	1.96 (1.12-3.-04)^c^	1.57 (0.75-2.60)^b^	<0.05
HOMA-β (%)	103.57 (58.29-168.33)^b^	107.89 (61.99-178.45)^a^	95.11 (50.53-162.86)	<0.05
Adjusted HOMA-β (%)	117.00 (70.00-170.50)^b^	128.00 (82.00-196.00)^a^	117.00 (74.00-164.00)	<0.05
QUICK I	0.61 (0.55-0.72)	0.62 (0.55-0.71)^c^	0.64 (0.56-0.79)^b^	<0.05
Adjusted QUICK I	0.55 (0.49-0.59)	0.55 (0.49-0.59)	0.56 (0.51-0.61)	0.062
Matsuda Index	85.53 (54.59-133.91)^b^	80.99 (52.76-136.74)^ac^	95.46 (50.39-150.05)^b^	<0.05
Adjusted Matsuda Index	114.18 (85.05-140.54)	105.31 (69.46-130.06)	116.22 (79.67-136.26)	0.053
CHOL (mmol/L)	4.94±0.88	5.05±0.91	4.95±1.00	0.489
TG (mmol/L)	1.45±1.39	1.31±0.72	1.39±0.98	0.478
HDL (mmol/L)	1.55±0.34	1.54±0.38	1.57±0.45	0.802
LDL (mmol/L)	2.87±0.82	3.02±0.84	2.87±0.76	0.172

DHEAS, dehydroepiandrosterone sulfate; AUCG area under the curve of glucose; AUCI, area under the curve of insulin; I/G, the ratio of AUCI and AUCG; Matsuda Index, Matsuda insulin sensitivity index.

a P<0.05 when compared with <45 days group.

b P<0.05 when compared with 45-90 days group.

c P<0.05 when compared with >90 days group.

### Comparison of Different Ovulatory Function in Women With Less Than 45 Days Bleeding Interval

A previous study found that approximately 10% of women with PCOS were ovulatory, although they had an irregular menstrual cycle (11). We further grouped women with less than 45 days of bleeding interval according to the ovulatory function and classified as less than 45 days ovulatory and less than 45 days anovulatory (**Table 3**). Compared with ovulatory women, those who were anovulatory showed a higher level of BMI and an increasing trend of LH/FSH, mFG, the glucose level of fasting, 1 and 2 h, fasting insulin, and 1 h blood insulin value, resulting in a relatively higher level of HOMA-IR, HOMA-β, and QUICK I but a decreasing trend of SHBG and Matsuda index (**Table 3**). Meanwhile, CHOL, TG, and LDL also tended to increase in anovulatory women but the level of HDL was decreased, suggesting that the ovulatory function of women with PCOS not only had an effect on glucose but also lipid metabolism.

**Table 3:** Comparison of ovulatory women with anovulatory women in <45 days group.

	<45 days		*P* value
	Ovulatory (N = 59)	Anovulatory (N = 82)	
BMI (kg/*m* ^2^)	22.15±3.37	23.20±4.87	<0.05
WHR	0.83±0.06	0.83±0.07	0.201
LH/FSH	1.38±1.04	1.58±1.69	0.273
T (mmol/L)	2.08±0.86	2.03±1.09	0.162
DHEAS (μg/dl)	2088.58±910.47	2050.90±900.67	0.865
AFC	25.82±7.05	24.21±6.90	0.643
FAI	3.89 (2.29-7.00)	3.74 (1.95-6.02)	0.734
mFG	3.00 (2.00-9.00)	3.50 (1.00-8.00)	0.308
SHBG (nmol/L)	55.91 (30.03-82.32)	50.99 (30.19-76.81)	0.525
Fasting glucose (mmol/L)	5.15±0.53	5.18±0.60	0.311
1h glucose (mmol/L)	7.90±2.53	8.36±2.59	0.551
2h glucose (mmol/L)	6.51±1.91	6.84±1.91	0.968
Fasting insulin (mU/ml)	7.76 (3.79-11.20)	8.54 (5.74-14.35)	0.064
1h insulin (mU/ml)	76.90 (49.50-108.00)	83.75 (52.03-136.00)	0.496
2h insulin (mU/ml)	68.70 (40.40-102.00)	65.35 (42.00-114.50)	0.915
AUCG	13.15 (11.60-15.55)	14.18 (11.68-16.49)	0.356
AUGI	121.25 (78.28-161.49)	124.70 (78.98-206.28)	0.630
I/G	8.33 (5.94-11.30)	8.98 (6.31-13.53)	0.715
HOMA-IR	1.64 (0.84-2.61)	1.96 (1.24-3.19)	0.087
HOMA-β (%)	92.42 (50.53-158.33)	119.00 (67.75-171.16)	0.124
QUICK I	0.64 (0.57-0.78)	0.61 (0.54-0.69)	0.087
Matsuda Index	96.50 (56.67-143.06)	82.03 (47.88-127.20)	0.179
CHOL (mmol/L)	4.89±0.90	4.96±0.87	0.583
TG (mmol/L)	1.39±1.73	1.50±1.07	0.844
HDL (mmol/L)	1.61±0.36	1.51±0.31	0.063
LDL (mmol/L)	2.81±0.94	2.91±0.73	0.170

### Comparison of Different Androgen Level in Women With 45 Days Longer Bleeding Interval

Apart from IR, hyperandrogenism is considered as another important pathological mechanism of PCOS. We further divided women with 45 days longer bleeding interval into the non-HA group and HA group (**Table 4**). Women with HA of both 45–90-day and 90-day-longer vaginal bleeding intervals showed a higher BMI, although there was no significance in the latter group. Similar results were also observed in women with HA with higher levels of T, DHEAS, FAI, mFG, glucose and insulin level of fasting, 1 and 2 h blood glucose and insulin values, HOMA-IR, and HOMA-β. Meanwhile, QUICK I and the Matsuda index showed a decreasing trend under the situation of HA. However, the change of lipid metabolism parameters was not as obvious as glucose, suggesting that the situation of HA may aggravate the disorder of glucose metabolism in women with PCOS (**Table 4**).

**Table 4:** Comparison of PCOS women with and without HA in 45-90 days and >90 days group.

	45-90 days		>90 days		*P* value
	Non-HA (N = 113)	HA (N = 151)	Non-HA (N = 37)	HA (N = 59)	
Age (y)	26.96±4.42^b^	25.12±5.37^a^	25.97±4.30	25.34±4.56	<0.05
BMI (kg/m^2^)	22.06±3.29	23.11±4.30^c^	20.87±3.04^bd^	23.26±4.64^c^	<0.05
WHR	0.82±0.07	0.81±0.06	0.82±0.07	0.81±0.08	0.768
LH/FSH	1.70±1.03^d^	1.68±0.99^d^	1.73±0.99	2.14±1.41^ab^	<0.05
T (mmol/L)	1.83±0.80^bd^	2.47±1.09^a^	2.14±1.04^d^	2.72±0.90^ac^	<0.05
DHEAS (μg/dl)	1873.61±884.21^bd^	2284.40±1089.47^a^	1995.42±1005.34	2391.19±1005.39^a^	<0.05
AFC	27.09±6.33	24.91±8.97^d^	27.06±6.10	28.46±11.29^b^	<0.05
FAI	3.09 (1.69-4.11)^bd^	6.33 (3.32-9.68)^ac^	2.64 (1.53-4.32)^bd^	6.17 (3.83-9.46)^ac^	<0.05
mFG	2.00 (1.75-3.00)^bd^	6.00 (3.00-10.00)^ac^	2.00 (1.00-3.00)^bd^	7.00 (4.00-9.75)^ac^	<0.05
SHBG (nmol/L)	61.27 (47.29-83.04)^bd^	35.68 (24.11-60.72)^ac^	87.27 (58.36-111.63)^bd^	47.87 (27.11-63.89)^ac^	<0.05
Fasting glucose (mmol/L)	5.08±0.40	5.13±0.60	4.91±0.43	5.10±0.51	0.138
1h glucose (mmol/L)	8.23±2.17	8.43±2.19	7.63±2.23	8.44±2.33	0.253
2h glu (mmol/L)	6.55±1.70	6.99±1.82^c^	6.11±1.17^b^	6.79±1.72	<0.05
Fasting insulin (mU/ml)	7.48 (3.90-9.81)	8.64 (5.56-15.40)^c^	4.15 (3.38-7.22)^b^	8.52 (4.36-13.28)	<0.05
1h insulin (mU/ml)	74.55 (47.83-127.50)^c^	87.20 (59.90-139.00)^c^	50.40 (41.80-74.40)^abd^	84.10 (59.63-145.00)^c^	<0.05
2h insulin (mU/ml)	60.45 (36.50-109.00)^b^	71.20 (47.50-141.00)^ac^	53.00 (39.30-72.70)^b^	71.60 (55.38-142.75)	<0.05
AUCG	13.10 (11.74-15.73)	13.60 (11.90-15.85)	12.95 (11.90-15.15)	14.95 (12.31-16.85)	0.136
AUGI	108.22 (75.21-185.74)^c^	128.48 (88.94-223.05)^c^	84.35 (67.48-116.36)^abd^	138.08 (90.54-229.18)^c^	<0.05
I/G	7.69 (5.78-12.91)	9.32 (6.48-13.99)^c^	7.28 (5.13-9.15)^b^	9.66 (6.84-13.99)	<0.05
HOMA-IR	1.64 (0.93-2.26)	1.92 (1.15-3.49)^c^	0.90 (0.78-1.57)^b^	1.98 (0.88-3.07)	<0.05
HOMA-β (%)	88.68 (49.75-139.10)	118.31 (65.89-204.62)^c^	58.71 (41.00-103.14)^b^	104.70 (58.80-178.40)	<0.05
QUICK I	0.64 (0.59-0.76)^c^	0.61 (0.53-0.77)^c^	0.77 (0.65-0.80)^ab^	0.61 (0.54-0.77)	<0.05
Matsuda Index	89.10 (59.31-155.46)^c^	76.84 (43.05-127.99)^c^	147.14 (93.65-177.31)^abd^	77.69 (42.95-126.05)^c^	<0.05
CHOL (mmol/L)	5.12±0.91	5.00±0.91	4.97±0.95	4.94±1.02	0.70
TG (mmol/L)	1.17±0.61	1.40±0.76	1.00±0.43^d^	1.51±1.08^c^	<0.01
HDL (mmol/L)	1.61±0.36	1.49±0.38	1.00±0.43	1.51±1.08	0.08
LDL (mmol/L)	3.07±0.80	2.99±0.86	2.78±0.85	2.90±0.0.73	0.38

a P<0.05 when compared with women in 45-90 days group without HA.

b P<0.05 when compared with women in 45-90 days group with HA.

c P<0.05 when compared with women in >90 days group without HA.

d P<0.05 when compared with women in >90 days group with HA.

## Discussion

In this study, we validated the severity of IR and the level of androgen was significantly higher in women with PCOS when compared with controls. Further analysis indicated a significant correlation between the degree of menstrual disturbance and the severity of IR by grouped women with PCOS according to the intervals between episodes of vaginal bleeding. A similar relationship between the LH/FSH ratio and the severity of IR was also observed. Meanwhile, HA may aggravate the disorder of glucose metabolism in PCOS.

A growing number of studies have shown that PCOS is often accompanied by IR, and obese PCOS subjects have a higher risk of developing IR (13, 19). Studies revealed that the incidence of IR in normal-weight PCOS patients is approximately 65%, while the incidence of IR is as high as 95% in obese PCOS subjects (14, 20). In our study, we found that women with PCOS had a significantly higher BMI than controls although the diagnostic criteria for obesity were not met (**Table 1**). Hyperinsulinemia caused by IR, HA, and changes in the paracrine signal of the follicle can interfere with the activation, growth, and selection of the follicle, and damage the normal development and ovulation of the follicle (21). Many studies have shown that IR may damage the development of oocytes and embryo quality, and is related to the low fertilization rate and implantation rate of women with PCOS (22–24). Identifying clinical markers that can effectively predict the occurrence of IR may help to improve the pregnancy outcome of women with PCOS. In our study, menstrual dysfunction, reflected by vaginal bleeding interval, was found to be correlated with the severity of IR, these results were consistent with previous studies (11, 25). Interestingly, women with PCOS and vaginal bleeding episodes of 45–90-day intervals had higher HOMA-IR and HOMA-β but lower QUICK I and Matsuda index than those with a 90-day-longer cycle, whereas the prevalence of delayed insulin peak went up with the prolongation of vaginal bleeding intervals. These observations may be related with the worse function of the islet B cell in PCOS subjects with vaginal bleeding intervals longer than 90 days.

Oligomenorrhea or an irregular menstrual cycle is reported to be associated with higher androgen levels and a lower level of SHBG (9). We found that with the prolongation of the vaginal bleeding intervals, the testosterone levels in PCOS patients gradually increased. Previous studies have shown that the serum androgen levels in both healthy women and those with PCOS are positively correlated with the number of follicles, and anti-androgen therapy in women with PCOS can effectively improve the situation of polycystic ovarian morphology (26, 27). There is a hypothesis that HA is the first hit to the development of PCOS follicles. High androgen levels in follicles promote the recruitment of small follicles, and excessively recruited small follicles will inhibit the selection process of dominant follicles (28, 29). These studies suggest that HA in PCOS patients may disrupt the menstrual cycle by impairing the normal development of follicles. In the subgroup analysis of this study, we noticed that women with HA of both 45–90 days and 90 days longer vaginal bleeding intervals had a worse situation of IR, indicating that HA may aggravate the severity of IR in PCOS. These results were similar to our previous study, in which HA was proven to be independently associated with the risk for obesity and type 2 diabetes (30). Then, a more severe IR induced a continuous excessive production of androgen (31, 32).

Although there are several studies that proved the positive relationship between menstrual disturbance and the severity of IR, the internal mechanism needs further study. PCOS subjects with IR were reported to exacerbate abnormal steroid production in granulosa cells, which is related with anovulation (33). A systemic glucose uptake disorder is considered as one of the main features of IR (34). GLUT4, expressed in insulin-sensitive tissues, is the main protein responsible for insulin-mediated glucose transport to adipocytes (35, 36), playing an important role in regulating glucose tolerance, glucose metabolism, and insulin sensitivity (37). A previous study found that the expression level of GLUT4 in the endometrial epithelial cells of PCOS subjects with HA was significantly lower than that of the control group (38), and GLUT4 dysfunction may lead to the occurrence of IR in PCOS (39, 40). The dysregulation of GLUT4 expression in granulosa cells may be the inner link between IR and oligomenorrhea, which is an underlying mechanism worthy to explore.

In summary, we found that the abnormality of androgen metabolism in women with PCOS changed with the severity of oligomenorrhea, and the severity of IR in women with PCOS is positively correlated with the vaginal bleeding interval. The menstrual disturbance reflected by the length of a menstrual cycle may be an effective indicator to predict IR in women with PCOS.

## Data Availability Statement

The raw data supporting the conclusions of this article will be made available by the authors, without undue reservation.

## Ethics Statement

The studies involving human participants were reviewed and approved by the Ethics Committee of Sun-Yat Sun Memorial Hospital of Sun Yat-Sen University. The patients/participants provided their written informed consent to participate in this study.

## Author Contributions

XL: data collection, data analysis, and manuscript writing. DYY: manuscript writing. PP: data collection. RA: project development. DZY: project development. YC: project development. XZ: project development and manuscript writing. All authors contributed to the article and approved the submitted version.

## Funding

This work was supported by the GDPH Supporting Fund for Talent Program (KY012021439), the National Key Research and Development Program of China (2017YFC1001004), National Natural Science Foundation of China (81771545), the Guangdong Basic and Applied Basic Research Foundation (2020B1515020001), and the Special Fund for Clinical Research of Chinese Medical Association (18010180747).

## Conflict of Interest

The authors declare that the research was conducted in the absence of any commercial or financial relationships that could be construed as a potential conflict of interest.

## Publisher’s Note

All claims expressed in this article are solely those of the authors and do not necessarily represent those of their affiliated organizations, or those of the publisher, the editors and the reviewers. Any product that may be evaluated in this article, or claim that may be made by its manufacturer, is not guaranteed or endorsed by the publisher.
